# Substance Abuse and Excessive Mortality Among Forensic Psychiatric Patients: A Finnish Nationwide Cohort Study

**DOI:** 10.3389/fpsyt.2019.00678

**Published:** 2019-09-13

**Authors:** Ilkka Ojansuu, Hanna Putkonen, Markku Lähteenvuo, Jari Tiihonen

**Affiliations:** ^1^Department of Forensic Psychiatry, Niuvanniemi Hospital, Kuopio, Finland; ^2^Addiction Psychiatry, University of Helsinki and Helsinki University Hospital, Helsinki, Finland; ^3^Institute for Molecular Medicine Finland (FIMM), University of Helsinki, Helsinki, Finland; ^4^Department of Clinical Neuroscience and Center for Psychiatry Research, Karolinska Institutet, Stockholm City Council, Stockholm, Sweden; ^5^Department of Forensic Psychiatry, University of Eastern Finland, Kuopio, Finland

**Keywords:** forensic psychiatry, mental illness, substance abuse, mortality, accidental death

## Abstract

**Background:** Forensic psychiatric patients are known to have reduced life expectancy. The aim of this study was to explore to what extent substance abuse disorders account for this increased mortality.

**Methods:** Data up to December 31, 2016 for mortality (causes of death register) and substance abuse (forensic psychiatric examinations) were collected for all of the 950 patients committed to involuntary forensic psychiatric hospital care in Finland during 1980–2009 and discharged no later than December 31, 2016. Patients were then classified as suffering or not suffering from substance abuse disorders and their causes of death were examined. The standardized mortality ratio was then calculated for these groups on the basis of sex-, age-, and calendar-period-specific mortality rates for the general Finnish population.

**Results:** During the follow-up time (mean 13.4 years), 354 (320 men, 34 women) patients died, resulting in a standardized mortality ratio of 3.5. The standardized mortality ratio for the patients with a history of substance abuse disorders was 4.1 compared to 2.8 for those with no such history. Among men, but not women, the age-adjusted proportion of death was significantly higher for those with a history of substance abuse disorders. In addition, in patients with a history of substance abuse disorders, the male age-adjusted competing risk of mortality was higher for unnatural causes, but not natural causes. Furthermore, a prominent proportion (16%) of all deaths and a majority of the accidental deaths (64%) occurred under the influence of some substance.

**Conclusions:** Substance abuse is a major factor causing excessive mortality among forensic psychiatric patients. The management of substance abuse problems should be one cornerstone of the treatment of patients with both severe mental disorders and substance abuse disorders during their time in hospital and this should be extended to outpatient care.

## Introduction

All major psychiatric disorders are associated with an increased risk of premature mortality (1). The mortality of patients discharged from a psychiatric hospital has been found to be four-fold higher than the general population in a Finnish sample (2). The mortality associated with substance abuse disorders has been found to be even higher than that associated with serious psychiatric disorders, like schizophrenia, schizoaffective disorder, or bipolar disorder (3, 4). This risk for premature mortality seems, at least to some extent, to be additive, as patients with both comorbid substance abuse and serious mental disorders are at an even higher risk (5, 6, 7).

It is not surprising that the mortality of forensic psychiatric patients, who often suffer from both serious psychiatric disorders as well as substance use disorders (SUDs), is higher than that of the general population. In a Swedish sample, the mortality of forensic psychiatric patients was found to be higher, if the primary diagnosis was that they were suffering from a substance abuse disorder (8). This was also the case for forensic patients with a secondary diagnosis of a substance abuse disorder, unless they were suffering primarily from bipolar disorder. However, only 34% of the Swedish sample consisted of patients diagnosed with schizophrenia or some other psychotic disorder. This sample included patients from many decades, and it need be noted that the average treatment time of the sample was stated to have been only 5 months, which might not reflect current practice. In Finland, in order to treat individuals as forensic psychiatric patients, they are required to have a diagnosis of a psychotic disorder and the average treatment times are on average many years (9). In a Finnish study, the mortality among forensic psychiatric patients was found to be up to three-fold higher than the general population, but somewhat comparable to that of other schizophrenia spectrum patients (4, 9). When the causes of death were further examined, most of the deaths in the forensic psychiatric patients were due to somatic illnesses, although the largest difference, i.e., as much as seven-fold elevated risk, was attributable to suicide (10).

The problem in extrapolating these data for forensic patients from country to country is that the criteria for placing an individual into forensic psychiatric care vary between countries, as do treatment practices, even though in general, the psychiatric treatment protocols might be similar. Thus, results from different countries might not be generalizable, unless these criteria and their treatment protocols are similar. There is a rather sparse literature on the effect of substance abuse disorders on mortality in forensic psychiatric patients with psychotic disorders. It is evident that a more detailed knowledge of the factors behind the increased mortality observed in forensic psychiatric patients is needed to guide treatment decisions towards reducing these substantial risks.

The aim of this study was to explore the extent to which substance abuse disorders contribute to the increased mortality observed in forensic psychiatric patients, even when treatments times, and thus periods of abstinence, are long. This information would be of major clinical interest, since there is a dogma surrounding many addictive disorders that the time of abstinence itself is a protective factor against relapse and further that relapse is a risk factor for increased mortality. If a long period of abstinence *per se* is not sufficient to prevent relapses for substance abuse disorders, then it is clear that there is a need to devise alternative treatment modalities for patients with substance abuse problems in forensic psychiatric hospitals.

## Materials and Methods

In Finland, the law court decides whether it is necessary to perform a forensic psychiatric examination which assesses the criminal responsibility of a defendant. Usually defendants committing homicides, or individuals who, due to their medical history or behaviour in detention, are thought to be affected by a psychiatric disorder, are subjected to a forensic psychiatric examination. After the forensic psychiatric examination, if the defendant is assessed as suffering from a serious mental disorder (psychosis or other disorders that affect reality testing, but not intellectual disability, autism or SUD by themselves), he/she can be exempted from legal punishment and be committed to involuntary forensic psychiatric hospital care. In the final stage of hospital treatment, the patient can be released on supervised leave, although he/she will still be under involuntary care. A supervised leave may be granted for up to 6 months at a time; furthermore, there can be multiple supervised leaves before ultimate hospital discharge. Most of the forensic psychiatric patients undergo this form of supervised leave prior to their final hospital discharge. After the patient’s ultimate discharge from hospital, psychiatric outpatient care is not mandatory; in legal terms, ex-forensic psychiatric patients are regarded in the same manner as other psychiatric outpatients. Finnish legislation does not allow for compulsory or involuntary outpatient care for any psychiatric patient. The Finnish National Institute for Health and Welfare (THL) is responsible for both the initiation and termination of involuntary psychiatric forensic hospital care.

### Data Acquisition

The material for this study was collected from the Finnish National Institute for Health and Welfare’s archive, which houses data on all Finnish forensic psychiatric examinations and the information of patients who have been committed to or released from involuntary forensic psychiatric hospital care. The data from patients which constituted this study group were then linked to the national cause of death register of Statistics Finland, which contains information on all deaths in Finland, including data on causes of and events related to death, which made it possible to estimate mortality. Standard mortality data for the general population, to be used as control data, were also retrieved from this register.

### Analyses

Our study population consisted of the 950 patients who had been committed to involuntary forensic psychiatric hospital care in Finland during the 30-year period from 1980 to 2009 and were discharged no later than 31.12.2016 (total number of patients committed during this time was 1,253). Follow-up started on hospital discharge and ended either on 31.12.2016 or when the patient died, whichever came sooner. The data for initial diagnoses (for psychosis and SUDs) were recorded from the forensic psychiatric examinations, which were then further screened for signs of SUDs not recorded in the diagnoses section, since the primary function of the examinations is to provide information on the individual’s mental state (e.g., psychotic symptomology) and substance abuse disorders are sometimes omitted from the diagnoses section as they may be thought to either be secondary to the evaluation or to have arisen from the psychotic disorder. These data were pooled together to classify a patient as suffering or not suffering from an SUD according to ICD-10 criteria. Sometimes, it proved difficult to ascertain enough information to determine whether the criteria for addiction had been fulfilled, although harmful use was clearly evident. Patients with current unequivocal evidence of harmful use or addiction were classified as having an SUD, regardless of which substance was being abused (ICD-10: F1x.1 - F1x.2). Those patients for whom there was only evidence of intoxication or withdrawal symptoms without a longer standing substance abuse disorder, patients with only prior evidence of SUDs without current use, or patients without any evidence of an SUD were classified as not having an SUD.

Data on causes of death and events related to death from the patients were then retrieved up to 31.12.2016 from the cause of death register, and the causes of death were then categorized as being due to somatic diseases, suicides, accidents, homicides, or unclear. If signs of substance use at time of death or prior to death were evident from the death certificates, these were also recorded.

The Standardized Mortality Ratio (SMR) was then calculated for all patients, grouping the patients as either suffering or not suffering from SUD as described above. The SMR was calculated as the ratio of observed and expected number of deaths by using subject-years methods with 95% confidence intervals, assuming a Poisson distribution. The expected number of deaths was calculated on the basis of sex-, age-, and calendar-period-specific mortality rates in the general Finnish population. We used Cox proportional hazards model to calculate the age adjusted hazard ratios HR for death and adjusted survival (failure) function. A competing-risks regression model (Fine and Gray model) with a robust estimate of variance served to estimate the adjusted subhazard ratios (sHR) and cumulative incidence in the presence of competing risks. Stata 15.0 (StataCorp LP, College Station, TX, USA) statistical package was used for the analysis.

### Ethical Considerations and Approval

This study is a part of the transnational After Care project. Ethics committee approvals for the study were sought and obtained from the Research Ethics Committees of Kuopio, Oulu and Turku Universities, Kuopio, Helsinki and Turku University Hospitals, Healthcare Centre of the City of Helsinki, Hospital District of Southern Savo, and the Hospital District of Pirkanmaa. This study was also approved by and the study material gathered from the Finnish National Institute for Health and Welfare and Statistics Finland. This study was registry based, and no contact was made with the study subjects.

## Results

There was a total of 950 forensic patients detected and included in the analyses. All of the patients were diagnosed as suffering from a psychotic disorder; the majority of them had a schizophrenia spectrum disorder, more specifically 59% had schizophrenia (ICD-10: F20.x), 13% had delusional disorder (F22.x), 9% had schizoaffective disorder (F25.x), and the rest other psychiatric disorders affecting reality testing, such as severe bipolar disorder, psychotic depression, organic brain injuries, or severe borderline personality disorder. The vast majority (823 = 86.6%) of the 950 forensic psychiatric patients were men and 127 (13.4%) were women. The mean duration of forensic psychiatric treatment had been 6.7 years [standard deviation (SD) 5.5], and the mean age of the patient was 43 years (SD 13) at the time of his/her discharge. The mean follow-up time was 13.4 years (SD 9.3 years). In Finland, a substance abuse disorder in itself is not sufficient grounds for treatment as a forensic psychiatric patient, but the majority (567 = 59.7%) of the patients (514 men, 53 women) were noted as suffering from a comorbid SUD according to ICD-10 criteria (either addiction or harmful use) in conjunction with their psychotic disorder. Of these 567 patients with an SUD, 395 were diagnosed prior to or during the forensic psychiatric examination; the other 172 were classified as having an SUD according to the examination notes of the forensic psychiatrist, even though no official diagnoses had been set for them.

During the follow-up, a total of 354 patients died. The mean follow-up time for these patients was 10.3 years (SD 8.1), resulting in a SMR of 3.5 for the whole patient population. The vast majority of deaths (264 = 74.6%) were attributable to somatic diseases; 80 (22.6%) of the deaths were due to unnatural causes (accidents, suicides, homicides); and in 10 (2.8%) cases, the cause of death had remained undefined even after forensic autopsy and had therefore been classified as unclear in their death certificate.

Most, 320, of the 354 deceased patients were men and 34 were women. Among these deceased patients, 218 were noted as having a substance abuse disorder during their forensic psychiatric examination. The SMR for the patients with an SUD was 4.1, whereas the SMR for the patients without an SUD was 2.8.

**Figure 1:**
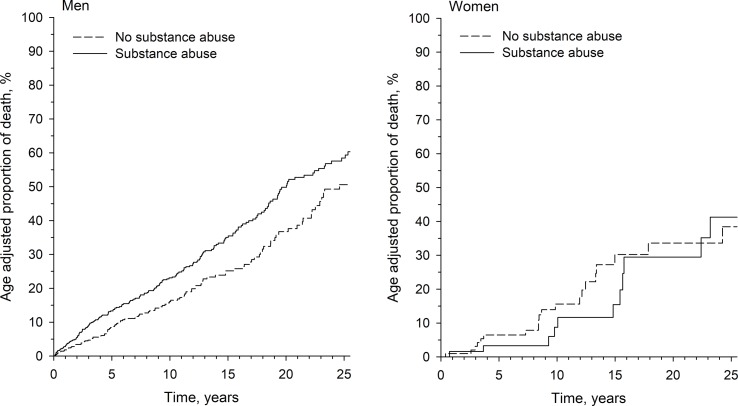
Age-adjusted proportions of death in percentages as a function of follow-up time for men and women with or without a substance use disorder.

Among men, the age-adjusted proportion of death was significantly higher among those with an SUD when compared to those without this disorder [hazard ratio (HR) = 1.34, 95% confidence interval (95% CI) 1.07 to 1.69, p = 0.012], but this kind of difference was not observed among women (HR = 1.00, 95% CI 0.50 to 2.01, p = 0.99). The age adjusted proportions of death are shown in **Figure 1**.

**Figure 2:**
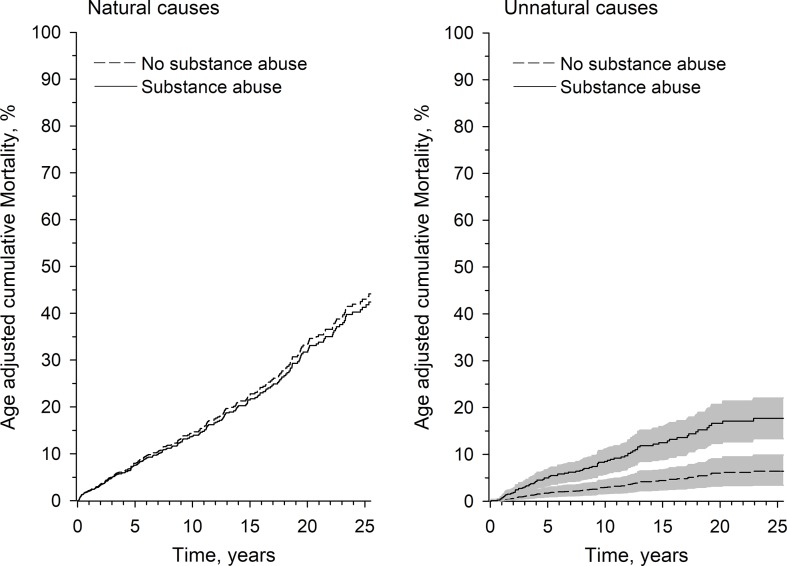
Age-adjusted competing risk of mortality in percentages as a function of follow-up time in men with or without a substance use disorder for both natural and unnatural causes. The figure for unnatural death displays hazard ratios (lines) and confidence intervals (shadings around lines).

The age-adjusted competing risk of mortality among men with a known SUD was not higher for the risk of death due to diseases (sHR 0.95, 95% CI 0.73 to 1.24, p = 0.70), but was significantly higher for the risk of dying from unnatural causes (sHR 2.63, 95% CI 1.55 to 4.47, p = 0.015). The competing mortality risks for men are shown in **Figure 2**.

Many of the examined death certificates mentioned that the current status of substance use preceding death was not known, though in many of the cases even when a background of substance use was recognized, the relationship between substance use and death remained somewhat obscure. However, in 56 of the 354 deaths, there was clear evidence of current substance use listed in the death certificates, such as evidence of intoxication or withdrawal symptoms at time of death or the fact that the subject had been found deceased with items for substance use, such as needles and syringes or alcohol. Of these 56 deceased, 47 had a history of SUD, only 9 did not (Chi^2^ statistic 14.04, p > 0.001 for history of an SUD vs. evidence of substance use at time of death, **Table 1**). Deaths related to current substance use with regard to history of an SUD are presented in **Table 1**. The numbers of deaths with/without current evidence of substance use, subdivided into causes of death, and the percentage of patients with or without clear evidence of current substance use at time of death are presented in **Table 2**.

**Table 1:** Number of deaths related to current substance use with regard to history of substance use disorder (Chi^2^ statistic 14.04, p > 0.001).

Chi^2^	Deaths related to current substance use	Deaths unrelated to current substance use
History of SUD	47	171
No history of SUD	9	127

**Table 2:** Number of deaths with/without current evidence of substance use subdivided into causes of death and by gender. The percentage of patients with or without clear evidence of current substance use at time of death is given in parentheses.

	Men	Women	Total
Suicide – substance use related –substance use unrelated	3 27	0 3	33 – 3 (9%) – 30 (91%)
Accident – substance use related – substance use unrelated	27 14	1 2	44 – 28 (64%) – 16 (36%)
Homicide – substance use related – substance use unrelated	1 2	0 0	3 – 1 (33%) – 2 (67%)
Unclear – substance use related – substance use unrelated	5 5	0 0	10 – 5 (50%) – 5 (50%)
Disease – substance use related – substance use unrelated	18 218	1 27	264 – 19 (7%) – 245 (93%)

If one assesses the natural deaths, then the cause of death was stated to have been directly caused by substance abuse in 10 patients; in more specific terms, three had alcohol related liver cirrhosis, three had alcohol related heart disease, one had combined alcohol related liver cirrhosis and heart disease, one had alcohol dementia, and for two, the main cause of death had been listed as SUD.

The majority (28/44 = 64%) of the unnatural deaths due to accidents were substance related. Fifteen of these deaths were attributable to substance overdoses or poisonings, 10 of which were alcohol intoxications. Of the remaining 13 substance related accidental deaths, four individuals had choked on food, two had died of subdural hemorrhage, one had frozen to death, one had drowned, one had died due to carbon monoxide poisoning, and one had suffocated after passing out in an awkward position, all while intoxicated. Three deaths happened during the withdrawal stage: one patient had frozen to death, one died of a subdural hemorrhage, and one due to clozapine poisoning after an extended period of drinking.

In 10 cases, the classification of death was unclear; for example, in some cases, it could not be determined whether a blunt force trauma was due to an accident or suicide, but in 5 of these cases, there was evidence of substance use.

## Discussion

Our results show that even after a long period of abstinence due to institutionalized forensic psychiatric care, especially men with a history of substance abuse disorders, in comparison with their counterparts without such a background, still have a significantly elevated risk for premature mortality after their release from care, especially due to unnatural causes. The same phenomenon was not observed in women, but this might be due either to actual gender-related differences or possibly due to the small sample size of our female study population. Furthermore, many of the deaths observed in the patient groups actually occurred while the patient was under the influence of substances, indicating obvious relapses of their substance abuse disorder.

As compared to previous studies investigating this topic, the strengths of this study are the large sample of forensic psychiatric patients, the prolonged mean follow-up time of 13.4 years, and the possibility to access the comprehensive and validated Finnish national registers (11). One weakness of this study was that we were unable to obtain information on the living arrangements, medication use and commitment to outpatient care, inpatient care episodes, or criminal convictions after discharge from the hospital. There was also no information available on what kind of treatment, if any, patients had received for their SUDs in addition to the forced abstinence during their hospital incarceration. Also, as only patients with clear evidence of current SUDs from the forensic psychiatric examinations were classified as having an SUD, some patients with marked substance use, but not clearly reaching diagnostic thresholds as assessed from the examination statements, were classified as not having an SUD, which serves to dilute the results presented here. It need also be noted that some 30% of the patients with clear diagnostic evidence of an SUD were left without a diagnosis of such in the initial forensic psychiatric mental state examinations, which could indicate a serious defect in recognizing substance abuse disorders and even possibly led to failure to provide proper treatment for them. It is also worth noting that knowledge of possible relapses to substance use was only available for those patients in whom it was mentioned in their death certificates. Thus, it is possible, perhaps even likely, that the overall rates of substance use relapses were higher. Some of the patients in the “no prior history of substance abuse” group might also have developed SUDs during their follow-up time, i.e., the present findings may be an underestimation. The data are thus subject to confounding by indication. In addition, as the current study is an observational registry based study, the data presented are only correlations, and only speculations of causality can be made.

In this study, the presence of substance abuse was found to contribute to mortality in the background of some somatic diseases, but it was especially evident in the large proportion of deaths due to accidents. Thus, although substance abuse might not be the only problem responsible for poor coping in some individuals, it is likely to be a major factor causing excessive mortality among forensic psychiatric patients due to both natural and unnatural causes. Therefore, the management of substance abuse disorders should receive a high priority in this patient group, in an attempt to reduce the excessive mortality as well as gaining other health benefits associated with reduced substance use. When viewed against the background of the long psychiatric hospital treatment provided for these patients, these results must be viewed both as a sign that abstinence in itself is not sufficient to prevent relapses and able to reduce the excessive mortality, but also as an indication of a failure to provide treatment modalities with greater efficacies.

## Conclusion

According to our study, a history of substance abuse is related to the excessive mortality observed in Finnish forensic psychiatric patients. Thus, the integrated management of addiction problems should be one cornerstone of the treatment of patients with both severe mental disorders and substance abuse disorders not only during their time in hospital but also extended to their outpatient care.

## Data Availability

The data analyzed in this study were obtained from Finnish national registers as described above. Due to the confidential nature of patient data, the data are not publicly available.

## Ethics Statement

The studies involving human participants were reviewed and approved by the Research Ethics Committees of Kuopio, Oulu and Turku Universities, Kuopio, Helsinki and Turku University Hospitals, Healthcare Centre of the City of Helsinki, Hospital District of Southern Savo, and the Hospital District of Pirkanmaa. Written informed consent for participation was not required for this study in accordance with the national legislation and the institutional requirements.

## Author Contributions

IO, HP, ML, and JT contributed to the conception and design of the study. IO organized the database and wrote the first draft of the manuscript. ML contributed to drafting and writing of the report. All authors contributed to manuscript revision and read and approved the submitted version.

## Funding

The study was funded by the Ministry of Social Affairs and Health, Finland, through the developmental fund for Niuvanniemi Hospital. The funder of the study had no role in study design, data collection, data analysis, data interpretation, or writing of the report.

## Conflict of Interest Statement

IO has received honoraria from Ratiopharm, consultancy fees from Camurus, and has attended a congress trip subsidized by MSD. ML is an owner and board member of Genomi Solutions Ltd, a Finnish-based bioinformatics company and has received honoraria or study grants from Sunovion Ltd, Orion Ltd, and the Finnish Medical Association. JT has participated in research projects funded by grants from Janssen-Cilag and Eli Lilly to his employing institution. JT reports lecture fees from Eli Lilly, Janssen-Cilag, Lundbeck, and Otsuka; consultancy fees from EMA (European Medicines Agency), Fimea (Finnish Medicines Agency), and Lundbeck; and grants from The Stanley Foundation and the Sigrid Jusélius Foundation. 

The remaining author declare that the research was conducted in the absence of any commercial or financial relationships that could be construed as a potential conflict of interest.
